# Effects of multilevel posterior ligament dissection after spinal instrumentation on adjacent segment biomechanics as a potential risk factor for proximal junctional kyphosis: a biomechanical study

**DOI:** 10.1186/s12891-018-1967-0

**Published:** 2018-02-14

**Authors:** Tobias Lange, Tobias L. Schulte, Georg Gosheger, Albert Schulze Boevingloh, Raul Mayr, Werner Schmoelz

**Affiliations:** 1 Department of Orthopedics and Tumor Orthopedics, Muenster University Hospital, Albert-Schweitzer-Campus 1, 48149 Muenster, Germany; 2 Department of Orthopedics and Trauma Surgery, St. Josef-Hospital, University Hospital, Ruhr-University Bochum, Gudrunstrasse 56, 44791 Bochum, Germany; 3 Department of Trauma Surgery, Medical University of Innsbruck, Anichstrasse 35, 6020 Innsbruck, Austria

**Keywords:** Proximal junctional kyphosis, Posterior instrumentation, Posterior ligaments, Ligament dissection, Biomechanics

## Abstract

**Background:**

Spinous processes and posterior ligaments, such as inter- and supraspinous ligaments are often sacrificed either deliberately to harvest osseous material for final spondylodesis e.g. in deformity corrective surgery or accidentally after posterior spinal instrumentation. This biomechanical study evaluates the potential destabilizing effect of a progressive dissection of the posterior ligaments (PL) after instrumented spinal fusion as a potential risk factor for proximal junctional kyphosis (PJK).

**Methods:**

Twelve calf lumbar spines were instrumented from L3 to L6 (L3 = upper instrumented vertebra, UIV) and randomly assigned to one of the two study groups (dissection vs. control group). The specimens in the dissection group underwent progressive PL dissection, followed by cyclic flexion motion (250 cycles, moment: + 2.5 to + 20.0 Nm) to simulate physical activity and range of motion (ROM) testing of each segment with pure moments of ±15.0 Nm after each dissection step. The segmental ROM in flexion and extension was measured. The control group underwent the same loading and ROM testing protocol, but without PL dissection.

**Results:**

In the treatment group, the normalized mean ROM at L2-L3 (direct adjacent segment of interest, UIV/UIV + 1, PJK-level) increased to 104.7%, 107.3%, and 119.4% after dissection of the PL L4–L6, L3–L6, and L2–L6, respectively. In the control group the mean ROM increased only to 103.2%, 106.7%, and 108.7%. The ROM difference at L2-L3 with regard to the last dissection of the PL was statistically significant (*P* = 0.017) and a PL dissection in the instrumented segments showed a positive trend towards an increased ROM at UIV/UIV + 1.

**Conclusions:**

A dissection of the PL at UIV/UIV + 1 leads to a significant increase in ROM at this level which can be considered to be a risk factor for PJK and should be definitely avoided during surgery. However, a dissection of the posterior ligaments within the instrumented segments while preserving the ligaments at UIV/UIV + 1 leads to a slight but not significant increase in ROM in the adjacent cranial segment UIV/UIV + 1 in the used experimental setup. Using this experimental setup we could not confirm our initial hypothesis that the posterior ligaments within a long posterior instrumentation should be preserved.

## Background

Posterior instrumentation of the spine is one of the most frequently performed surgical procedures for various pathologies. According to a report by the Agency for Healthcare Research and Quality, approximately 488.000 spinal fusions were performed during U.S. hospital stays in 2011 (a rate of 15.7 stays per 10.000 population) [1]. In spinal deformity corrective surgery complication rates vary from 37 to 68% [2]. One of the major complications is proximal junctional kyphosis (PJK) with a varying incidence of 6 to 61.7% [3–7]. The rate of revision surgery due to PJK can be as high as 50% [5]. Numerous risk factors for PJK have been described in the literature, including:

- disruption of the posterior ligaments (supra- and interspinous ligaments, PL) and facet capsules [8]
- the type, stiffness, and combination of the implants selected [9–15]; wedging of the disc above or below an instrumentation [16]
- vertebral compression fractures in the upper instrumented vertebra (UIV) or the first proximal adjacent vertebra [17]
- the choice of the UIV in deformity correction [3, 18] and the influence of the sagittal parameters [19].

However, neither the precise pathophysiology of PJK nor clear strategies for preventing it have yet been established [3, 19].

One of the keys to PJK is the quality and integrity of the posterior tension banding [3, 8, 12]. This includes the supra- and interspinous ligaments. Many surgeons keep the spinous process of the UIV, the PL between the UIV and UIV + 1, as well as the facet joint capsules between UIV and UIV + 1 intact but sacrifice the spinous processes and PL caudal to the UIV, especially in deformity corrective surgery. The objective of this procedure is to facilitate a better deformity correction e.g. by Ponte osteotomies and to harvest osseous material which is used for the final spondylodesis.

The purpose of this biomechanical in vitro study was to evaluate the potential destabilizing effect of a progressive dissection of the PL, even within the fused segments caudal to UIV following posterior instrumentation on the segmental biomechanics at UIV/UIV + 1. It was hypothesized, that the PL even within the fused segments caudal to UIV are relevant for sagittal stability, that a dissection could therefore lead to an increase of range of motion (ROM) at UIV/UIV + 1 and could possibly represent a risk factor for the development of PJK and that the PL should therefore be preserved during surgery.

## Methods

### Specimen preparation

Twelve L1–L6 calf lumbar spines (aged 12–18 months) obtained from a local slaughterhouse were used according to established biomechanical testing protocols [15, 20, 21]. The specimens were stored at − 20 °C and thawed overnight at 4 °C before testing. All soft tissue was removed, leaving the PL, capsules, and other supporting ligamentous structures intact. The specimens were embedded in polymethylmethacrylate (PMMA; Technovit 3040, Heraeus Kulzer, Wehrheim, Germany) at the upper half of the L1 and the lower half of the L6 vertebrae. They were mounted in a well-established spine tester [22, 23] with the middle disc (L3–L4) aligned horizontally. Screws for fixation of the three-dimensional motion analysis system (Winbiomechanics, Zebris Medical Ltd., Isny, Germany) were fixed to the ventral side of the PMMA blocks and vertebrae L2, L3, and L4.

Posterior instrumentation at L3–L6 was performed by an experienced spine surgeon using a bilateral polyaxial pedicle screw rod system (screws: 6.0 × 45 mm for the L3, L4, and L5 vertebrae, 7.0 × 45 mm for L6; rod: titanium, diameter 5.5 mm; Expedium® System, DePuy Synthes, Raynham, MA, USA).

The specimens were randomly assigned to one of the two study groups (treatment vs. control group). The tests were performed at room temperature, and the specimens were kept moist with physiological saline solution during testing.

### Biomechanical test setup

In the treatment group, six specimens underwent stepwise dissection of PL (supra- and interspinous ligaments, from caudal to cranial) within the fused segments and at last also at UIV/UIV + 1. After each level of ligament dissection (four states: intact, PL L4–L6, PL L3–L6, and PL L2–L6), the specimens underwent cyclic flexion motion of 250 cycles in which a flexural moment of + 2.5 to + 20.0 Nm was applied (triangular loading function, 2.5°/sec) to simulate physical activity. The number of load cycles was limited to 250 cycles due to time constraints of in vitro testing with cadaveric specimens (maximal one day of testing before degradation sets in). The load magnitudes were chosen rather high to provoke an effect of stretching ligamentous structures beyond the elastic region to cause an increased motion in the segment to simulate the genesis of PJK. This was followed by a flexibility test with pure moments of ±15.0 Nm in flexion/extension for three load cycles measuring the ROM of each segment.

**Fig. 1:**
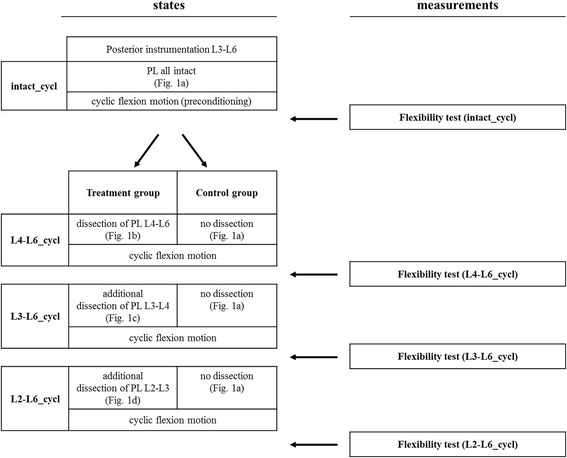
The sequence of the experiment. For each state, flexibility tests were carried out after cyclic flexion motion, and the data obtained were used for statistical analysis

**Fig. 2:**
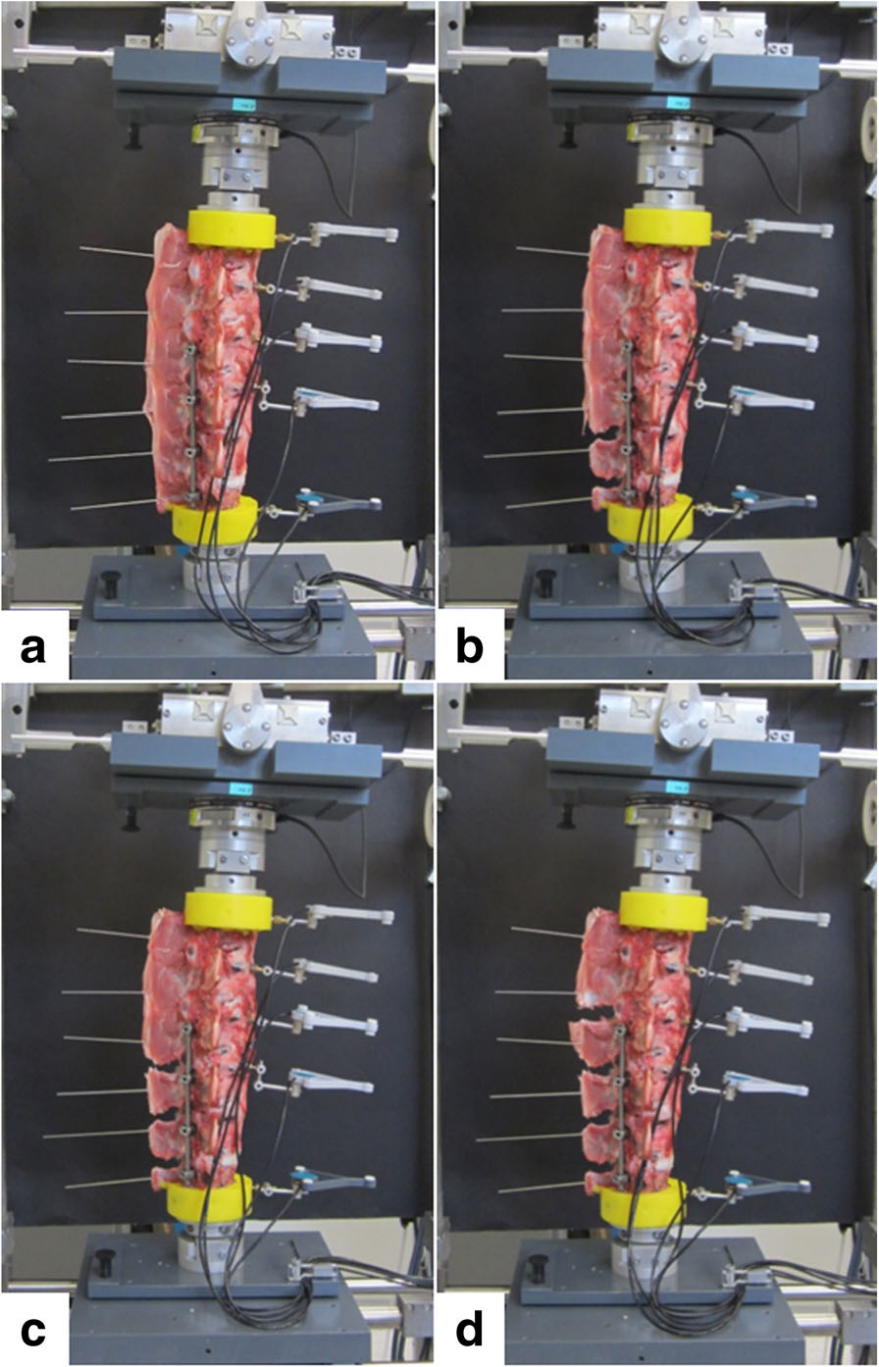
States of the specimens during testing in the treatment group. **a** Instrumented (intact). **b** Dissection of supraspinous and interspinous ligaments L4–L6. **c** Additional dissection of supraspinous and interspinous ligaments L3–L4. **d** Additional dissection of supraspinous and interspinous ligaments L2–L3

In the control group, six specimens underwent the same test protocol with cyclic motion and flexibility tests, but without PL dissection (Figs. 1 and 2).

**Fig. 3:**
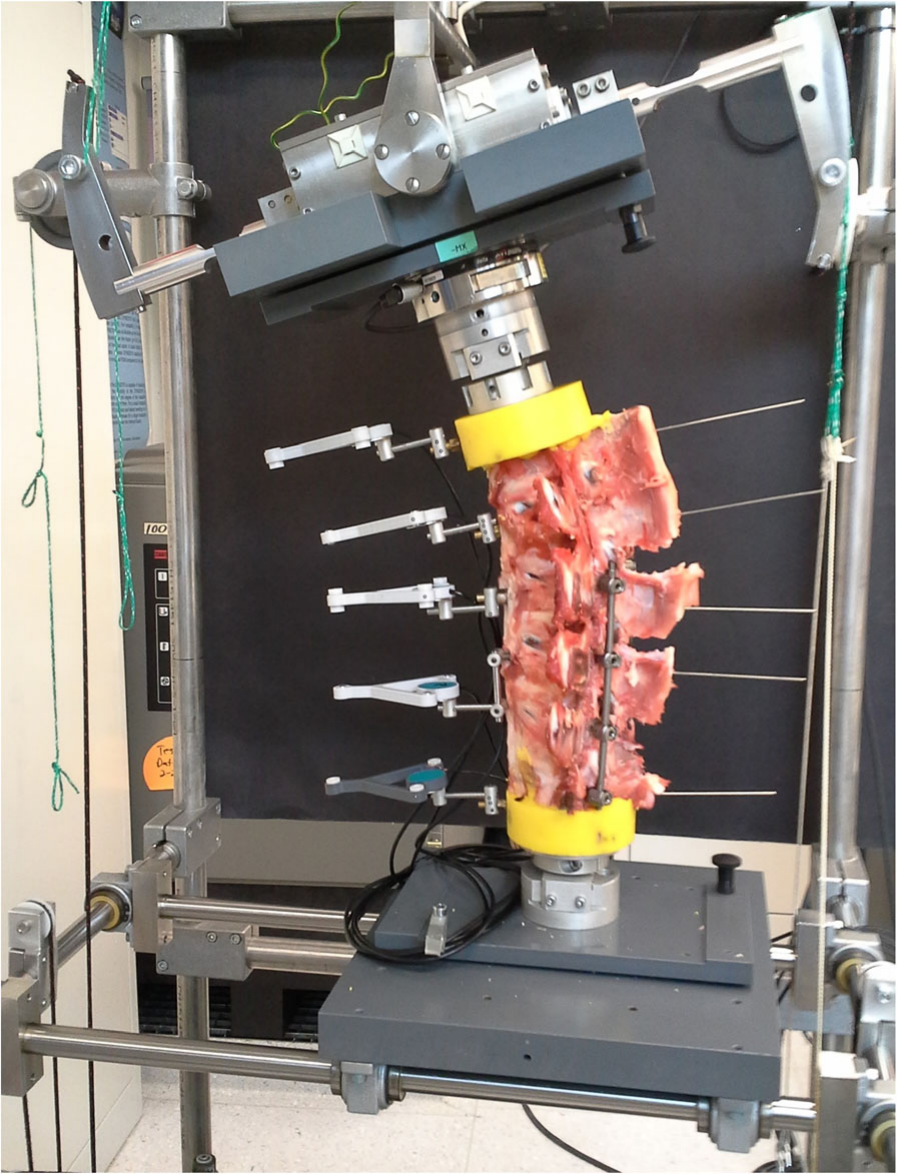
Six degrees of freedom spine simulator for in vitro biomechanical testing

Flexibility tests and cyclic flexion motion were done in the same setup of a six degree of freedom spine tester. The specimens were loaded with pure moments, applied by a stepper motor. A six-component load cell with feedback control was connected to the stepper motor to control the loading of the specimens (Fig. 3). No preload or specific preconditioning was applied to the specimens. The test speed was 0.7°/sec. Intersegmental motions were measured using an ultrasound-based motion analysis system. From the recorded data, the ROM was measured during the third load cycle for segments L1–L2, L2–L3, L3–L4, and L4–L6.

### Data evaluation

After instrumentation, a baseline flexibility test was performed. To facilitate data comparison, subsequent ROM measurements were normalized to the initial ROM. Statistical analysis was performed using IBM SPSS Statistics® for Windows, version 22.0 (IBM Corporation, Armonk, New York). For data comparison analysis, two-way analysis of variance (ANOVA) with repeated measures was performed (four states: intact_cycl, PL L4–L6_cycl, PL L3–L6_cycl, and PL L2–L6_cycl) with post hoc analysis using Bonferroni correction. To account for possible sphericity violation among the states, *P* values were corrected using the Greenhouse–Geisser method [24]. The significance level was set at *p* < 0.05.

## Results

The ROM for each segment (L1-L2, L2-L3, L3-L4, L4–L6) was measured for each state (intact_cycl, PL L4–L6_cycl, PL L3–L6_cycl, PL L2–L6_cycl) (Table 1).

**Table 1:** Absolute values of range of motion (ROM)

Segment	State	Treatment group		Control group	
		Mean (°)	SD (°)	Mean (°)	SD (°)
L1–L2	intact_cycl	9.88	3.68	9.51	1.18
	L4-L6_cycl	10.44	3.97	9.83	1.25
	L3-L6_cycl	10.73	4.07	10.29	1.23
	L2-L6_cycl	11.25	4.22	10.56	1.34
L2–L3	intact_cycl	10.47	2.56	11.27	1.65
	L4-L6_cycl	10.97	2.74	11.64	1.69
	L3-L6_cycl	11.24	2.83	12.02	1.74
	L2-L6_cycl	12.50	3.47	12.25	1.70
L3–L4	intact_cycl	1.53	0.73	1.71	0.26
	L4-L6_cycl	1.60	0.72	1.75	0.25
	L3-L6_cycl	1.66	0.75	1.82	0.24
	L2-L6_cycl	1.71	0.76	1.85	0.24
L4–L6	intact_cycl	2.58	0.81	3.16	0.84
	L4-L6_cycl	2.81	0.87	3.30	0.85
	L3-L6_cycl	2.87	0.89	3.43	0.83
	L2-L6_cycl	2.84	0.80	3.47	0.80

Absolute values of range of motion (ROM) in degrees during testing of segments L1–L2, L2–L3, L3–L4, and L4–L6 in the treatment and control groups

**Fig. 4:**
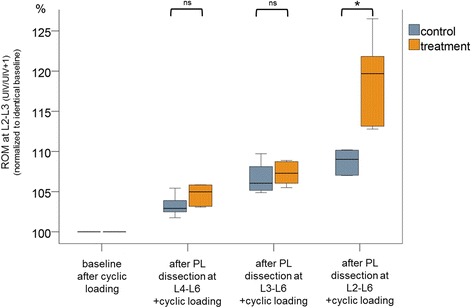
Changes in range of motion (ROM) at segment L2–L3 (UIV/UIV + 1) after stepwise dissection of the posterior ligaments (L4–L6, L3–L6, L2–L6), normalized to the initial flexibility test as basis (intact_cycl) in percentages, after preconditioning by cyclic loading. * Significant at *P* < 0.05, ns: not significant

The most important approval of this study is that a dissection of the PL including L2-L3 (UIV/UIV + 1) leads to a significant increase of ROM at the adjacent segment L2-L3 (UIV/UIV + 1) itself, whereas dissection of PL within the instrumented segments (keeping PL of UIV/UIV + 1 intact) results in a slight but not significant increment of ROM at the segment L2-L3 (UIV/UIV + 1) (Fig. 4).

In the treatment group, the normalized mean ROM in segment L2-L3 (direct adjacent segment of interest, UIV/UIV + 1, PJK-level) increased to 104.7%, 107.3% and 119.4% after dissection of the L4–L6, L3–L6 and L2–L6 PL, respectively (Fig. 4). In the control group, the mean ROM increased only to 103.2%, 106.7% and 108.7%, respectively, without dissections for each state. The difference in the L2-L3 segment with regard to changes in ROM across testing states between the two groups was statistically significant (*p* = 0.017). The interaction between treatment (dissection or control) and state (intact, PL L4–L6, PL L3–L6 and PL L2–L6) was also statistically significant (*p* = 0.002).

The normalized mean ROM of segment L1-L2 (second adjacent segment, UIV + 1/UIV + 2) increased to 105.5%, 108.6%, and 113.9% after dissection of the L4–L6, L3–L6, and L2–L6 PL, respectively (Fig. 4) in the treatment group. Whereas in the control group, an increase to 103.3%, 108.3%, and 111.0% was seen after cyclic flexion motion without dissections for each state. The differences between the two groups with regard to changes in ROM across the testing states were not significant (*p* = 0.154). The interaction between treatment (dissection or control) and state (intact, PL L4–L6, PL L3–L6, and PL L2–L6) was also not significant (*p* = 0.171).

The segments L4-L6 and L3-L4 (segments within the rigid posterior instrumentation) show only small absolute values of ROM (Table 1) and they do not show any significant changes within the treatment group after PL dissection (L4-L6: *p* = 0.496, L3-L6: *p* = 0.245) compared to the native state. The reason is the rigid posterior instrumentation spanning from L3-L6.

## Discussion

This study shows that a dissection of the posterior ligaments (the supraspinous and interspinous ligaments) at UIV/UIV + 1 does have a significant influence on the ROM in the adjacent segment and is therefore definitely a risk factor for PJK.

PJK is defined as pathologic kyphosis in the first mobile proximal adjacent segment after instrumentation. Whereas some authors define PJK radiographically as kyphosis greater than 10° between the upper instrumented vertebra and the vertebral body two levels above it (UIV/UIV + 2) [4], others require this angle to be additionally at least 10° greater than the corresponding preoperative measurement [25]. According to Arlet and Aebi, PJK is a junctional kyphosis of 15° or more above a previous instrumentation [3]. Various failure modes have been proposed [3]: progressive deformity above a previous instrumentation, representing the natural course of the deformity; wedging of the disc above or below an instrumentation [16]; vertebral compression fractures in the upper instrumented vertebra or the first proximal adjacent vertebra [17]; failure of proximal fixation (screw pull-out, screw ploughing, screw cutting into end plates); dislocation of the spine above the instrumentation; disc degeneration in the adjacent segment; and in particular, elongation with or without disruption of the PL [8].

The incidence of PJK is reported to be in the range of 6–61.7% [3–6]. In most cases, PJK develops during the first 3 months after surgery [25], and this may represent evidence for failure of the posterior ligament complex intraoperatively or immediately postoperatively.

Various risk factors have been suggested in the literature as being responsible for PJK. Some of these cannot be controlled or influenced by the surgeon, but others can [3, 8, 26–28]. One of the key factors that can be influenced by the surgeon is the amount of tissue disruption [3, 8]. In addition to the facet joint capsules at the UIV + 1/UIV level and the PL are relevant posterior tension banding structures. The practical question during surgery is whether to sacrifice spinous processes in the upper part of the instrumentation or whether to keep them — and thus the ligamentous tension banding — intact. The ligaments distal to the spinous process of the UIV and also the spinous processes of UIV-1 and below are often resected in order to harvest material for posterior spondylodesis. During surgery, a sudden loss of tension in the adjacent inter- and supraspinous ligaments was occasionally observed during dissection of supraspinous and interspinous ligaments, even far away within the instrumentation. It is a fact that the fibers of the supraspinous ligament are attached multisegmental to the spinous processes [29] and it was therefore hypothesized that these ligaments and spinous processes distal to the UIV are relevant for sagittal stability and should be preserved in order to prevent PJK.

Cahill et al. have shown in a finite element modeling an increased angular displacement and nucleus pressure immediately after dissection of the posterior ligamentous complex at the level above the construct [8]. Different dissection procedures of the facet joints and ligaments at the level UIV + 1/UIV have been analyzed already by Cammarata et al., they carried out a virtual biomechanical analysis of PJK using computer simulation and also showed that dissection of the PL, bilateral facetectomy, and a combination of the two between UIV and UIV + 1 lead to increases in the proximal junctional angle of 10%, 28%, and 53%, respectively [12]. Liu et al. have also shown protective effects of a preservation of the posterior complex (complete laminectomy vs. hemi-laminectomy vs. facet joint resection only) on the development of adjacent segment degeneration after lumbar fusion [28]. Additionally, Pham et al. proposed an application of a tendon graft as an interspinous ligament reinforcement between UIV + 1 and UIV-2 as a preventive strategy for proximal junctional kyphosis [30].

However, there is no previous biomechanical study with an experimental setup evaluating the effect of a dissection of the posterior ligaments even within the instrumented levels, far away from the junction level, and not only at the adjacent level itself. Therefore, we conducted this biomechanical study which at least supports the theory that there is an important tension band effect of the PL preventing PJK in the adjacent segment. In our study a small, nonsignificant increase in ROM at UIV/UIV + 1 was detected after PL dissection even within the fused segments of the spine. Most notably, a PL dissection at UIV/UIV + 1 itself has a significant impact on the ROM in this segment (+ 18.9%) and therefore on the potential development of PJK.

**Fig. 5:**
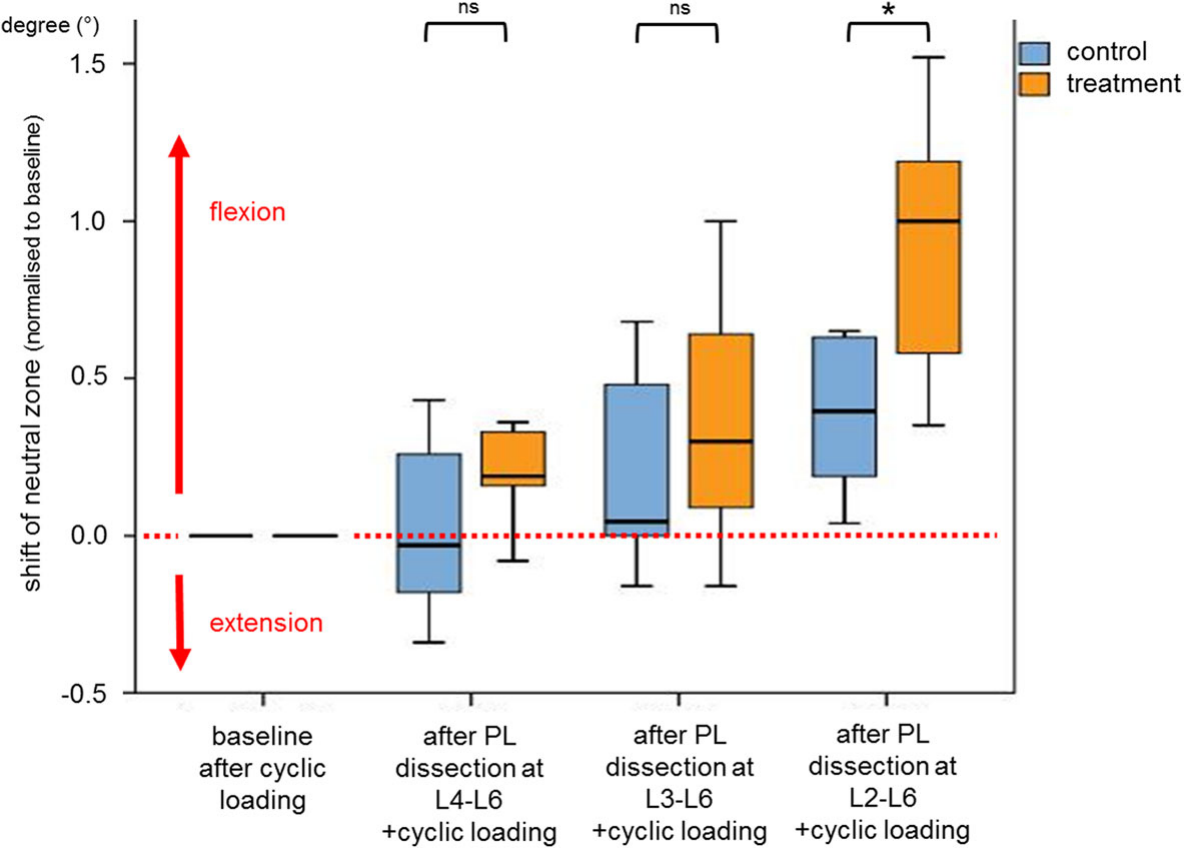
Changes in the mean neutral zone of the control and treatment group of the segment L2-L3 (UIV/UIV + 1). A change in the positive direction represents a shift towards kyphosis. * Significant at *P* < 0.05, ns: not significant

As an increase in ROM is only an indicator and not equal to an increased kyphosis, i.e. PJK, the hysteresis curves (applied moment vs resulting angular displacement) of the flexibility tests were further evaluated to emphasize the effect of posterior ligament dissection on segmental alignment. The ROM mainly increased in flexion, while the ROM in extension did not substantially change. With this the mean neutral zone as an indicator of the segmental alignment also shifted in the kyphotic direction. This became even more pronounced with an increasing dissection of the posterior ligaments (Fig. 5) and the difference between the control and dissection group became significant (*p* < 0.02) at the final dissection. To simulate a physical activity a cyclic flexion motion was performed in both groups. Therefore, all ligamentous structures and other tissues are strained and might lose their tension over time. This can explain the increase of the ROM in the treatment group as well as the small increase in the control group over time.

A limitation of the present study is the use of calf instead of human spines. These specimens do not represent the same sagittal profile as humans and therefore the absolute ROM values cannot be directly transferred to the situation in humans. However, the relative effects of the various states that were tested should be similar in humans. Previous studies have shown that the use of calf lumbar spines does allow accurate biomechanical studies comparable to human spines [20, 31]. The obvious difference between quadrupeds and humans is the everyday loading of the spine, while the human spine is loaded more in axial compressions whereas the spine of quadrupeds is subjected to more bending. It can therefore be assumed that there is an even more pronounced effect in human spines, which have greater thoracic kyphosis due to the upright body position. It might also be speculated that the spine in elderly humans is more susceptible to the development of PJK in comparison with younger spines, due to age-related tissue degenerative effects [32]. In this study, nonkyphotic lumbar spines were used. As PJK is mainly present in the thoracic spine, with its natural precondition of kyphosis, it may be speculated that the effect of an increase in ROM in the adjacent segment that is demonstrated here is even more distinctive in the kyphotic thoracic spine. We are well aware that we are not able to control the influence of sagittal parameters on the risk of development of PJK in this test setup. Even in case of sagittal parameter evaluation it could be speculated that these data would be of limited value as our setup is a bovine in vitro model.

Obviously, the biomechanical experiments were carried out with a limited number of specimens. However, due to the controlled laboratory environment the common confounding variables occurring in a clinical trial can be excluded. Therefore it can be assumed that if the biomechanical effect of an intervention cannot be shown in a controlled laboratory environment with a limited sample size, it is deemed to be unlikely to have a clinical impact.

Furthermore a more extensive cyclic flexion motion after each PL dissection simulating a higher and longer physical activity could have been resulted in an even more increased ROM at UIV/UIV + 1 coming to statistical significance, especially for the ligament dissection within the instrumented segments and should be evaluated in the future.

## Conclusions

Dissection of posterior ligaments (supraspinous and interspinous ligaments) of the adjacent segment (UIV/UIV + 1) cranial to a posterior instrumentation leads to a significant increase in ROM in the adjacent segment itself. Therefore, an accidental injury to or an intentional dissection of the posterior ligaments at UIV/UIV + 1 could be a risk factor for PJK and should definitively be avoided.

Dissection of the posterior ligaments within the instrumented segments while preserving the ligaments at UIV/UIV + 1 leads to a slight but not significant increase in ROM in the adjacent cranial segment UIV/UIV + 1 in the used experimental setup. Using this experimental setup we could not confirm our initial hypothesis that the posterior ligaments within a long posterior instrumentation should be preserved.

Further clinical evaluation is needed to finally answer the question of the role of the posterior ligaments regarding sagittal stability after posterior instrumentation.

## Abbreviations

e.g.: for example
PJK: Proximal junctional kyphosis
PL: Posterior ligaments
PMMA: Polymethylmethacrylate
ROM: Range of motion
UIV + 1: one vertebra above the upper instrumented vertebra
UIV: Upper instrumented vertebra
